# Long COVID 19 Syndrome: Is It Related to Microcirculation and Endothelial Dysfunction? Insights From TUN-EndCOV Study

**DOI:** 10.3389/fcvm.2021.745758

**Published:** 2021-11-30

**Authors:** Salma Charfeddine, Hassen Ibn Hadj Amor, Jihen Jdidi, Slim Torjmen, Salma Kraiem, Rania Hammami, Amine Bahloul, Nesrine Kallel, Nedia Moussa, Imen Touil, Aiman Ghrab, Jamel Elghoul, Zineb Meddeb, Yamina Thabet, Samir Kammoun, Kamel Bouslama, Sami Milouchi, Salem Abdessalem, Leila Abid

**Affiliations:** ^1^Cardiology Department, Hedi Chaker University Hospital Sfax, Sfax, Tunisia; ^2^University of Medicine of Sfax, University of Sfax, Sfax, Tunisia; ^3^Department of Cardiology, Tahar Sfar Hospital Mahdia, Sfax, Tunisia; ^4^Department of Preventive Medicine, Hedi Chaker University Hospital Sfax, Sfax, Tunisia; ^5^Department of Pneumology, Hedi Chaker University Hospital Sfax, Sfax, Tunisia; ^6^Department of Pneumology, Tahar Sfar Hospital Mahdia, Mahdia, Tunisia; ^7^Department of Cardiology, Habib Bourguiba Hospital Medenine, Sfax, Tunisia; ^8^Department of Pneumology, Habib Bourguiba Hospital Medenine, Sfax, Tunisia; ^9^Department of Internal Medicine, Mongi Slim LaMarsa Hospital, Tunis, Tunisia; ^10^Clinic Pasteur Tunis, Tunis, Tunisia

**Keywords:** COVID-19, long COVID-19 syndrome, endothelial function, microcirculation, endothelium

## Abstract

The COVID-19 disease is a multisystem disease due in part to the vascular endothelium injury. Lasting effects and long-term sequelae could persist after the infection and may be due to persistent endothelial dysfunction. Our study focused on the evaluation of endothelial quality index (EQI) by finger thermal monitoring with E4 diagnosis Polymath in a large cohort of long COVID-19 patients to determine whether long-covid 19 symptoms are associated with endothelial dysfunction. This is a cross-sectional multicenter observational study with prospective recruitment of patients. A total of 798 patients were included in this study. A total of 618 patients (77.4%) had long COVID-19 symptoms. The mean EQI was 2.02 ± 0.99 IC_95%_ [1.95**–**2.08]. A total of 397 (49.7%) patients had impaired EQI. Fatigue, chest pain, and neuro-cognitive difficulties were significantly associated with endothelium dysfunction with an EQI <2 after adjustment for age, sex, diabetes, hypertension, dyslipidemia, coronary heart disease, and the severity of acute COVID-19 infection. In multivariate analysis, endothelial dysfunction (EQI <2), female gender, and severe clinical status at acute COVID-19 infection with a need for oxygen supplementation were independent risk factors of long COVID-19 syndrome. Long COVID-19 symptoms, specifically non-respiratory symptoms, are due to persistent endothelial dysfunction. These findings allow for better care of patients with long COVID-19 symptoms.

## Introduction

The severe acute respiratory syndrome coronavirus 2 (SARS-CoV-2) was detected in China in December 2019 (1, 2). Up to the time of writing, more than 149 million people worldwide have been infected, and over 3 million people have died from the coronavirus disease 2019 (COVID-19) (3). The COVID-19 disease is a multisystem disease due to in part the vascular endothelium injury it causes (4, 5). Lasting effects and long-term sequelae could persist after the infection, affecting patients' return to work and quality-of-life (6). The most prevalent ongoing symptoms are fatigue, dyspnea, chest pain, joint pain, palpitations, anosmia and dysgeusia, hair loss, cognitive symptoms, and psychosocial distress (6–11). Some studies suggest that Long-COVID 19 symptoms may be due to persistent endothelial dysfunction (12). In fact, the SARS-CoV-2 infection of endothelial cells is associated with changes in cell morphology and endothelial cells apoptosis that could persist several weeks after the acute infection. The Working Group on Atherosclerosis and Vascular Biology together with the Council of Basic Cardiovascular Science of the European Society of Cardiology provided a Position Statement on the importance of the endothelial function study in convalescent patients for early detection and prevention of long-term cardiovascular complications (4).

Our study focused on endothelial function evaluation by finger thermal monitoring (FTM) of endothelial quality with an E4-diagnose device (Polymath Company) (13) in a large cohort of long COVID-19 patients.

The study objectives were defined as the assessment of long COVID 19 symptoms' prevalence, endothelial function in recovered COVID-19 patients, and its link to long COVID-19 symptoms.

## Materials and Methods

### Study Design

This was a cross-sectional multicenter observational study with prospective recruitment of patients. The recruiting period extended from January 20 to May 10, 2021. The study protocol was recorded in the Pan African Clinical Trials Registries (PACTR) with trial ID PACTR202102867544936. The study had the local Ethics and Investigation Committee approval, being designated with approval number CPP SUD 0299/2020.

### Participants

Patients were recruited by local health authorities relying on the COVID-19 registry. After being informed about the study and potential risks, all eligible patients–recovered from confirmed severe acute SARS-CoV2 infection and having given written informed consent–underwent a comprehensive non-invasive assessment of endothelial and cardiac function during their long-COVID19 infection period. Inclusion criteria were as follows: age >18 years, both sexes, written informed consent, and a recent diagnosis of COVID-19 infection in the past 2 weeks−6 months proven by RT-PCR analysis of nasopharyngeal swabs positivity or viral rapid test. Important exclusion criteria included: Diseases carrying a life-expectancy of <1 year according to clinical judgment, pregnancy and breastfeeding, and foreseen inability to attend scheduled visits.

The long COVID-19 symptoms were defined by persistent symptoms 4 weeks after the start of acute COVID-9 infection (14). These symptoms were assessed simultaneously with the microvascular circulation and endothelial function at the inclusion. An objective evaluation of reported symptoms was performed. Shortness of breath was defined by a New York Heart Association class of dyspnea ≥ 2 (15). Fatigue was evaluated according to the modified fatigue severity scale (16). We used the Mini-Mental State Examination (MMSE) to assess cognitive performances. Cognitive difficulties were defined by an MMSE score <24 (17).

### Test Methods

#### Assessment of Microvascular Circulation and Endothelial Function

All investigation sites used the E4-diagnose device (Polymath Company. Tunisia) with a fully automated and standardized post occlusion reactive hyperemia procedure (PORH). The E4-diagnose is a non-invasive, high resolution (0.002°C) skin temperature measuring device. It consists of a portable microcontroller (MCU) unit, two accurate finger temperature sensors, and an integrated wrist cuff. All the automated procedures and calculations are fully processed by the embedded MCU firmware. A dedicated PC software views, stores, and exports the data. The tests were carried out in a dimmed and quiet room. The ambient temperature (between 22–24°C) was maintained consistently during the test. The patient was fasting with no smoking nor heavy physical activity, for 4 h prior to the test. At least 20 min were allowed for acclimatization and subjects were kept in a relaxing sitting position. Systolic blood pressure was checked to be <160 mmHg and index fingers temperature above 27°C. The integrated wrist cuff is placed on the dominant forearm and both finger sensors are gently fixed to both index fingers.

The standard protocol is reported in Supplementary Material, Supplementary Figures 1, 2.

During TUN-EndCOV study, EQI was selected as the best parameter to reflect the endothelial function relying on the classification below:

- EQI ≥ 2: Good endothelial function
- EQI <2: Endothelial dysfunction

The cut-off of two was in accordance with reported previous data (18) and after statistical validation (Supplementary Material).

The group endothelial dysfunction was divided into two subgroups with a cut-off of 1.5 for further analysis.

#### Echocardiographic Evaluation

A complete echocardiographic evaluation of the systolic and diastolic left ventricle (LV) function was performed. The LV global longitudinal strain (LVGLS) was determined by the speckle tracking analysis.

### Statistical Analysis

Statistical analyses were performed using SPSS (Statistical Package for the Social Sciences) version 23.0. The complete database is maintained by the study team. Categorical variables were expressed as frequencies and percentages and continuous variables as mean and SD. Differences in percentages were assessed using the chi-2 test and their means using the Student's t-test. Estimates of risk ratios were presented with 95% Confidence Intervals (CI). All variables that were statistically significant at univariate analysis and those considered of relevant clinical interest with a risk of error of 10% were included in a multivariable model (logistic regression) to identify the independent predictors of endothelial dysfunction and long COVID 19 symptoms and to determine adjusted Odds Ratio (OR). Receiver operating characteristic (ROC) analysis was carried out to determine the cut-off value of continuous variables associated with endothelial dysfunction. A *p* <0.05 was considered statistically significant.

## Results

A total of 798 patients were included in this study. Patients were included at an average time of 68.934 ± 3.1 [28–186] days. The mean age was 49.94 ±14.2 years. Women accounted for 60.5 % of patients (483 of 798). Hypertension was the most common chronic health condition, reported in 33%, followed by diabetes in 23.7%. A total of 618 patients (77.4%) had long COVID 19 symptoms. The mean EQI was 2.02 ± 0.99 IC_95%_ [1.95–2.08]. A total of 397 (49.7%) patients had impaired EQI. The demographics, clinical characteristics, long-term medications, endothelial function, and echocardiographic parameters of the study population at inclusion according to the occurrence of long COVID 19 symptoms were reported in Table 1.

**Table 1:** Baseline characteristics of the study population.

		**Total population (*N* = 798)**	**Post COVID 19 symptoms (*N* = 618)**	**No post COVID 19 symptoms (*N* = 180)**	***p*-value**
**Clinical characteristics**					
Age (years)		49.94 ± 14.2	50.03 ± 14.2	49.65 ± 14.3	0.75
BMI (kg/m2)		28.34 ± 4.7	28.35 ± 4.6	28.32 ± 4.8	0.94
Females (*n*, %)		483 (60.5)	389 (62.9)	94 (52.2)	**0.01**
Diabetes (*n*, %)		189 (23.7)	153 (24.8)	36 (20.0)	0.18
Hypertension (*n*, %)		269 (33.7)	207 (33.5)	62 (34.4)	0.81
Dyslipidemia (*n*, %)		84 (10.5)	69 (11.2)	15 (8.3)	0.27
Smoking (*n*, %)		57 (7.1)	41 (6.6)	16 (8.9)	0.30
**CV risk factors**					
0		397 (49.7)	305 (49.4)	92 (51.1)	0.68
1		195 (24.4)	157 (25.4)	38 (21.1)	
2		130 (16.3)	89 (15.9)	32 (17.8)	
≥3		76 (9.5)	58 (9.4)	18 (10.0)	
Heart failure (*n*, %)		4 (0.5)	3 (0.5)	1 (0.6)	0.64
Coronary heart disease (*n*, %)		34 (4.3)	25 (4.0)	9 (5.0)	0.57
Pulmonary disease (*n*, %)		48 (6.0)	42 (6.8)	6 (3.3)	0.08
**Chronic medications before trial**					
Aspirin (*n*, %)		70 (8.8)	55 (8.9)	15 (8.3)	0.81
ACE inhibitors (*n*, %)		103 (12.9)	84 (13.6)	19 (10.6)	0.28
ARBs (*n*, %)		58 (7.3)	53 (8.6)	5 (2.8)	**0.008**
Bblockers (*n*, %)		84 (10.5)	56 (9.1)	29 (16.1)	**0.007**
Statins (*n*, %)		107 (13.4)	87 (14.1)	20 (11.1)	0.30
Calcium channel blockers (*n*, %)		37 (4.6)	29 (4.7)	8 (4.4)	0.88
Nitrates (*n*, %)		6 (0.8)	3 (0.5)	3 (1.7)	0.10
**Severity of COVID 19 infection**					
Moderate or severe symptoms (need of oxygen) (*n*, %)		185 (23.2)	159 (25.7)	26 (14.4)	**0.002**
**Extend of lesions at thoracic CT scan**					
≥50 % (*n*, %)		36 (4.5)	32 (5.2)	4 (2.2)	0.09
**Endothelial function parameters**					
EQI		2.02 ± 0.9	1.99 ± 0.9	2.09 ± 1.0	0.24
Flow_ratio		5.02 ± 3.2	5.02 ± 3.2	5.00 ± 3.3	0.93
Peak_time		51.86 ± 29.9	50.97 ± 28.5	54.95 ± 34.4	0.12
Half_time_decay		36.10 ± 17.5	35.74 ± 16.6	37.13 ± 19.9	0.42
**Endothelial dysfunction (EQI** ** <2)**		397 (49.7)	319 (51.6)	78 (43.3)	**0.05**
**Echocardiographic parameters**					
LVEF (%)		61.00 ± 5.3	60.94 ± 5.3	61.30 ± 5.1	0.65
LVGLS (%)		−16.96 ± 2.7	−16.84 ± 2.6	−17.63 ± 2.8	0.07
E-wave velocity (cm/sec)		79.33 ± 19.0	79.21 ± 19.2	79.91 ± 18.2	0.81
A-wave velocity (cm/sec)		74.15 ± 18.2	73.74 ± 18.5	76.11 ± 16.7	0.40
E' velocity (cm/sec)		13.81 ± 3.9	13.64 ± 3.7	14.57 ± 5.0	0.13
E/E'		6.09 ± 2.1	6.08 ± 2.0	6.14 ± 2.5	0.87
sPAP (mmHg)		21.64 ± 7.2	21.77 ± 7.4	21.06 ± 6.4	0.53
Elevated LV filling pressure (*n*, %)		2 (0.3)	1 (0.2)	1 (0.6)	0.4

*ACE, angiotensin-converting enzyme; ARB, angiotensin receptor blockers; BMI, body mass index; CV, cardiovascular; EQI, endothelium quality index; LVEF, left ventricular ejection fraction; LVGLS, left ventricular global longitudinal strain; E, transmitral early diastolic peak velocity; A, late diastolic peak velocity; E', early relaxation velocity on tissue Doppler; sPAP, systolic pulmonary artery pressure. Bold values mean statistically significant difference*.

Among long COVID-19 symptoms, fatigue was the most common symptom reported in 42.2%, followed by shortness of breath in 41.5%, headaches in 22.1%, and chest pain in 20.3%.

Long COVID-19 symptoms were associated with endothelium dysfunction with an EQI <2 (Table 2). Fatigue, chest pain, and neuro-cognitive difficulties were significantly associated with an EQI <2 after adjustment for age, sex, diabetes, hypertension, dyslipidemia, coronary heart disease, and the severity of acute COVID-19 infection (need for oxygen) (Table 2). Long COVID-19 symptoms were not associated with the severity of the endothelial dysfunction in the subgroups analysis.

**Table 2:** Adjusted associations of different long COVID 19 symptoms to endothelial dysfunction.

	**Total population (*n* = 798)**	**EQI ≥ 2 (*n* = 401)**	**EQI <2 (*n* = 397)**	***p*-value**	**OR_**95%**_**	Adjusted^*^ OR_**95%**_
≥ 1 symptom (*n*, %)	618 (77.4)	299 (74.5)	319 (80.3)	0.05	1.39 [0.99–1.39]	1.45 [1.02–2.07], *p* = 0.03
Chest pain, dyspnea or fatigue (*n*, %)	546 (68.4)	256 (63.8)	296 (73)	0.005	1.53 [1.13– 2.07]	1.50 [1.09-2.07], *p* = 0.01
Fatigue (*n*, %)	337 (42.2)	155 (38.7)	182 (45.8)	0.04	1.34 [1.01–1.78]	1.36 [1.01, 1.83], *p* = 0.038
Chest pain (*n*, %)	162 (20.3)	62 (15.5)	100 (25.2)	0.001	1.84 [1.29–2.62]	1.94 [1.34–2.80], *p* <0.001
Palpitations (*n*, %)	139 (17.4)	78 (19.5)	61 (15.4)	0.12	0.75 [0.52–1.08]	0.84 [0.57–1.24], *p* = 0.40
Shortness of breath (*n*, %)	331 (41.5)	159 (39.7)	172 (43.3)	0.29	1.16 [0.87–1.54]	1.12 [0.83- 1.52], *p* = 0.44
Cough (*n*, %)	136 (17)	63 (15.7)	73 (18.4)	0.31	1.20 [0.83–1.75]	1.15 [0.78–1.69], *p* = 0.47
Headaches (*n*, %)	176 (22.1)	86 (21.4)	90 (22.7)	0.67	1.07 [0.76–1.50]	1.26 [0.88–1.79], *p* = 0.19
Anosmia (*n*, %)	28 (3.5)	15 (3.7)	13 (3.3)	0.72	0.87 [0.40–1.85]	1.06 [0.47 – 2.35], *p* = 0.88
Gastro-intestinal syndrome (*n*, %)	47 (5.9)	18 (4.5)	29 (7.3)	0.09	1.67 [0.91–3.07]	1.62 [0.86–3.05], *p* = 0.13
Neuro-cognitive difficulties (*n*, %)	97 (12.2)	44 (11.0)	53 (13.4)	0.30	1.25 [0.81–1.91]	1.62 [1.03–2.55], *p* = 0.036
Sleep disorders	76 (9.5)	34 (8.5)	42 (10.6)	0.31	1.27 [0.79–2.05]	1.45 [0.88–2.40], *p* = 0.13

* *Adjusted to age, sex, diabetes, hypertension, dyslipidemia, coronary heart disease, and severe clinical status of COVID 19 infection with the need for oxygen.*

*EQI, endothelium quality index; OR, odds ratio*.

In multivariate analysis, endothelial dysfunction (EQI <2), female gender, and severe clinical status at acute COVID-19 infection with a need for oxygen supplementation were independent risk factors of long COVID-19 syndrome (Table 3).

**Table 3:** Associated factors to long COVID-19 syndrome in multivariate analysis.

**Variable**	**Odds Ratio, 95 CI%**	***P***
EQI <2	1.522 (1.072–2.160)	0.019
Female gender	1.913 (1.340–2.731)	<10^−3^
Severe clinical status of COVID 19 infection with need to oxygen	2.394 (1.495–3.833)	<10^−3^
B-blockers	0.489 (0.296–0.806)	0.005

*EQI, endothelium quality index*.

Endothelial dysfunction was significantly associated with the older age, body mass index (BMI), male gender, cardiovascular risk factors, the severity of symptoms during the acute phase of COVID-19 infection, the extension of pulmonary lesions during the COVID-19 infection, and reduced LV GLS (Table 4).

**Table 4:** Baseline characteristics in the study population according to endothelium quality index.

		**Total population (*n* = 798)**	**EQI ≥ 2 (*n* = 401)**	**Impaired EQI <2** **(*n* = 397)**	***p*-value**
**Clinical characteristics**					
Age (years)		49.94 ± 14.2	47.21 ± 14.5	52.71 ± 13.4	<10^−3^
BMI (kg/m^2^)		28.34 ± 4.7	27.55 ± 4.6	29.14 ± 4.6	<10^−3^
Females (*n*, %)		483 (60.5)	279 (69.6)	204 (51.4)	<10^−3^
Diabetes (*n*, %)		189 (23.7)	75 (18.7)	114 (28.7)	0.001
Hypertension (*n*, %)		269 (33.7)	112 (27.9)	157 (39.5)	0.001
Dyslipidemia (*n*, %)		84 (10.5)	26 (6.5)	58 (14.6)	<10^−3^
Smoking (*n*, %)		57 (7.1)	28 (7.0)	29 (7.3)	0.86
**CV risk factors**					
0		365 (45.7)	213 (53.1)	152 (38.3)	<10^−3^
1		223 (27.9)	108 (26.9)	115 (29.0)	
2		125 (15.7)	48 (12.0)	77 (19.4)	
≥3		85 (10.7)	32 (8.0)	53 (13.4)	
Heart failure (*n*, %)		4 (0.5)	1 (0.2)	3 (0.8)	0.31
Coronary heart disease (*n*, %)		34 (4.3)	10 (2.5)	24 (6.0)	0.01
Pulmonary disease (*n*, %)		48 (6.0)	26 (6.5)	22 (5.5)	0.57
**Chronic medications before trial**					
Aspirin (*n*, %)		70 (8.8)	19 (4.7)	51 (12.8)	<10^−3^
ACE inhibitors (*n*, %)		103 (12.9)	41 (10.2)	62 (15.6)	0.02
ARBs (*n*, %)		58 (7.3)	19 (4.7)	39 (9.8)	0.006
Bblockers (*n*, %)		84 (10.5)	34 (8.5)	50 (12.6)	0.05
Statins (*n*, %)		107 (13.4)	31 (7.7)	76 (19.1)	<10^−3^
**Severity of COVID 19 infection**					
Moderate or severe symptoms (need of oxygen) (*n*, %)		185 (23.2)	72 (18.0)	113 (28.5)	<10^−3^
**Extend of lesions at thoracic CT scan**					
≥50 % (*n*, %)		36 (4.5)	11 (2.7)	25 (6.3)	0.01
**Echocardiography**					
LVEF (%)		61.00 ± 5.3	61.93 ± 5.3	60.16 ± 5.1	0.004
LVGLS (%)		−16.96 ± 2.7	−18.19 ± 2.3	−15.89 ± 2.5	<10^−3^
E-wave velocity (cm/sec)		79.33 ± 19.0	83.49 ± 16.8	75.50 ± 20.1	<10^−3^
A-wave velocity (cm/sec)		74.15 ± 18.2	73.69 ± 17.5	74.58 ± 18.9	0.68
E' velocity (cm/sec)		13.81 ± 3.9	14.79 ± 4.0	12.9 ± 3.6	<10^−3^
E/E'		6.09 ± 2.1	5.95 ± 1.7	6.22 ± 2.4	0.28
sPAP (mmHg)		21.64 ± 7.2	20.97 ± 7.7	22.26 ± 6.7	0.13
Elevated LV filling pressure		2 (0.3)	0	2 (0.5)	0.24

*ACEI, angiotensin-converting enzyme inhibitors; ARBs, angiotensin receptor antagonists (ARBs); BMI, body mass index; CV, cardiovascular; EQI, endothelium quality index; LV, left ventricle, LVEF, left ventricular ejection fraction; LVGLS, left ventricular global longitudinal strain; E, transmitral early diastolic peak velocity; A, late diastolic peak velocity; E', early relaxation velocity on tissue Doppler; sPAP, systolic pulmonary artery pressure*.

According to ROC analysis, 45 years old, 25kg/m2, and−16% were the optimal cut-off values respectively for age, BMI, and LVGLS associated with endothelial dysfunction (Supplementary Figure 3). In multivariate analysis, age ≥ 45 years old, reduced LVGLS < -16%, and dyslipidemia were significantly associated with endothelial dysfunction (Table 5).

**Table 5:** Associated factors to endothelial dysfunction in multivariate analysis.

**Variable**	**Odds Ratio, 95 CI%**	***p***
Age > 45 years old	2.046 (1.151–3.635)	0.01
Reduced LVGLS	4.540 (2.451–8.410)	<10^−3^
Dyslipidemia	2.834 (1.042–7.713)	0.04

*BMI, body mass index; LVGLS, left ventricle global longitudinal strain*.

## Discussion

COVID-19 is a multisystem disease due to in part endothelium damage (4, 5). SARS-CoV-2 infects the host using the angiotensin-converting enzyme 2 (ACE2) receptors, which are expressed in several organs, including the lung, heart, kidney, intestine, and also expressed by endothelial cells, causing a distinguishable and distinct systemic endotheliitis (5, 19).

Lasting sequalae, symptoms, signs, or abnormal clinical parameters persisting 4 weeks or more after COVID-19 infection onset were commonly defined as “long COVID-19” (14, 20–22).

While it is well established that endothelial dysfunction is associated with poor prognosis in acute phase COVID19, its link with long COVID 19 symptoms is still questionable.

This study (1) reported long COVID-19 data in a large cohort of patients and (2) focused on endothelial dysfunction as a possible mechanism of underlying persistent long COVID-19 symptoms.

Our findings support that long COVID 19 syndrome is frequent. Recent reviews and metanalysis evaluated and summarized the best available evidence on the frequency of long COVID-19 (6, 11) (Table 6). Persistent long COVID-19 symptoms were reported until 6 months after the acute phase (23).

**Table 6:** Prevalence of long COVID-19 symptoms in the literature.

	**TUN-End-COV study**	**Systematic review and metanalysis of Lopez Leon et al. (6)**	**Systematic review of Cabrera Martimbianco et al. (11)**
At least one symptom (%)	77.4	80	4.7–80
Fatigue (%)	42.2	58	6.6–64.0
Chest pain (%)	20.3	16	0.4–89
Palpitations (%)	17.4	22	13
Shortness of breath (%)	41.5	24	5.5–61
Cough (%)	17	19	1.8–59.0
Headaches (%)	22.1	44	2.0–39.0
Anosmia (%)	3.5	-	0–26.2
Gastro-intestinal syndrome (%)	5.9	12	1.3–33.3
Neuro-cognitive difficulties (%)	12.2	43	18–57.1

Different risk factors such as old age, a high number of comorbidities, severe clinical status, hospital admission, and oxygen supplementation at the acute COVID-19 phase were reported as potentially associated with long COVID-19 symptoms (8, 10, 24–26). TUN-EndCOV study showed that persistent symptoms especially chest pain, fatigue, and neurocognitive symptoms (non-respiratory symptoms) during the long COVID-19 period were mainly associated with endothelial dysfunction, even after adjustment for age, sex, diabetes, hypertension, dyslipidemia, coronary heart disease, and severe clinical status of COVID-19 infection with the need for oxygen. In multivariate analysis, endothelial dysfunction was an independent risk factor of long COVID-19 syndrome. A recent case reported a non-amelioration of vascular reactivity 3 weeks after acute COVID-19 infection (27). A small-sized pilot study in patients with critical COVID-19 suggested that microvascular function assessed by Laser Speckle Contrast Imaging may not be fully recovered 3 months after disease onset (28).

The beta-blockers treatment was associated with reduced long COVID-19 symptoms. In fact, some papers suggested that beta-adrenergic blockers may be associated with beneficial effects during the acute COVID-19 infection by decreasing the SARS-CoV-2 virus entry, inhibiting NLRP3 inflammasome, reducing IL-6 and so that decreasing the consequent cardio-vascular and pulmonary acute COVID-19 complications (29). These mechanisms may also explain the long-term cardio-pulmonary COVID-19 symptoms. Furthermore, this benefit could be explained in part by beta-blockers pleiotropic effects on the endothelium (reduction of the myocardial oxygen consumption, and anti-oxidant properties) and their effect on rate control (30).

In this large cohort of long COVID-19 population, endothelial function was evaluated by an FTM of the endothelium quality with E4-diagnose device (Polymath Company. Tunisia) (13). It has been shown that the FTM can be used as a reproducible and operator-independent test for the non-invasive measurement of endothelial function in a controlled environment (31). In the largest report to date on any fingertip-based measurement of vascular reactivity and endothelial function, the vascular reactivity index (VRI) values were inversely correlated with age and male gender (18). Even so, the distribution of VRI values in the elderly population and between genders clearly showed a sizable number of good and intermediate scores (18). These findings support the clinical utility of FTM as a test that can differentiate good vascular function from poor vascular function, regardless of the characteristics of the patient (18). In the present study, older age and subclinical LV systolic dysfunction measured by LVGLS were an independently associated factor to impaired EQI. In previous studies, subclinical myocardial deformation with reduced LVGLS was reported at one-month follow-up in one out of every three patients recovered from COVID 19 infection, even in those without myocardial injury (32). The alteration of LVGLS associated with endothelial dysfunction following COVID-19 infection may be due to different factors: (1) the cardiomyocyte inflammation due to viral infiltration (33) and immune mechanisms (34, 35) (2) the hypoxia due to respiratory failure (34, 36), and (3) the myocardial injury due to microvascular dysfunction (19, 37, 38). This finding could explain the persistent chest pain during the long COVID-19.

This is the largest and the first study up to date that focused on the non-invasive evaluation of endothelial function by FTM during the long COVID-19. An important finding is the probable association of “non-respiratory” long COVID-19 symptoms especially chest pain, fatigue, and neuro-cognitive difficulties to endothelial dysfunction, highlighting the importance of an early evaluation of the endothelium quality for appropriate management. Understanding the pathophysiology and underlying mechanisms of persistent symptoms in long COVID-19 patients is required to guide investigations, management and improve patient prognosis.

Further studies are required to characterize long COVID-19 vascular sequelae in order to develop a planned monitoring program and adequate treatment.

### Limitations and Perspectives

One limitation of the study could be that reported symptoms were collected by a physician and not through self-reports, so probably only significant symptoms were reported. Furthermore, a global evaluation of the quality of life of the patients was not reported. Another study limitation could be the absence of data on whether patients with endothelial dysfunction had any subclinical preexistent endothelial dysfunction before COVID-19 infection especially in the elderly. In fact, age was one of the most important factors associated with endothelial dysfunction in our study. Longitudinal follow-up of individuals with long COVID-19 syndrome and endothelial dysfunction is warranted to better understand the pathophysiology underlying the long-term persistence and to guide therapeutic intervention. Randomized studies are requested to study the effect of treatment with action on the endothelium function such as Beta-blockers, ACE inhibitors, ARBs, and statins on the long COVID-19 symptoms. Finally, lack of control data regarding endothelial function evaluation in patients with similar demographic characteristics without COVID-19 infection could be one of the study limitations.

## Conclusion

There is increasing evidence regarding the link between endothelial dysfunction and persistent long COVID-19 symptoms. Risk stratification of long COVID-19 patients may be important to their management. The evaluation of endothelium quality by FTM to non-invasively detect endothelial dysfunction needs to be studied further to improve the management of long COVID-19 patients.

## Data Availability Statement

The raw data supporting the conclusions of this article will be made available by the authors, without undue reservation.

## Ethics Statement

The studies involving human participants were reviewed and approved by CPP SUD 0299/2020. Written informed consent for participation was not required for this study in accordance with the national legislation and the institutional requirements.

## Author Contributions

LA, SA, SC, and HI: conception and design of the study and literature review. JJ, SC, SA, and LA: analysis and interpretation of the data. SC: drafting of the manuscript. LA and SA: revising and editing the manuscript. All authors: data collection.

## Conflict of Interest

The authors declare that the research was conducted in the absence of any commercial or financial relationships that could be construed as a potential conflict of interest.

## Publisher's Note

All claims expressed in this article are solely those of the authors and do not necessarily represent those of their affiliated organizations, or those of the publisher, the editors and the reviewers. Any product that may be evaluated in this article, or claim that may be made by its manufacturer, is not guaranteed or endorsed by the publisher.
