# Supplementary material for: Long COVID 19 Syndrome: Is It Related to Microcirculation and Endothelial Dysfunction? Insights From TUN-EndCOV Study
Source: Front Cardiovasc Med. 2021 Nov 30;8:745758. doi: 10.3389/fcvm.2021.745758 (PMC8670225; doi:10.3389/fcvm.2021.745758)
Supplement: Supplementary file 1 [file Data_Sheet_1.docx]

Supplementary Material

# Supplementary Data

The standard protocol consists of three 300 seconds phases thus the whole test lasts 15 min:

- Initial phase: room, right and left index finger’s temperatures are sensed each second until reaching a steady state. Realtime skin perfusion blood flow (ml/mn/100g) is processed by the embedded firmware for both fingers. A 60 s seconds average and area under the curve are calculated and we get Flow0 and Flow0_AUC.
- Occlusion phase: the wrist cuff is automatically and rapidly inflated to 200 mmHg and kept at this pressure for 5 minutes. During this phase, the temperature of the occlusion side index will drop by 2 to 3°C following a cooling slope (Slope1). the blood flow curve will be at a near zero-perfusion level. During this phase, it is assumed the prolonged hand ischemia will cause a distal microvascular relaxation.
- Reperfusion phase: The wrist cuff is immediately deflated; the temperature of the occlusion side index rises immediately (Slope2). It is assumed that this slope depends on the peripheral microvascular dilation and the brachial, radial and cubital artery flow mediated dilation. During this phase many parameters are given (**Supplementary figure S1**):
- **Endothelium quality index (EQI)** is a dimensionless value comparing both slopes and curves of the occlusion and reperfusion phases.
- Peak_flow and **peak_time** are respectively the maximum blood flow reached and its timing since the reperfusion time index.
- **Half_time_decay:** is the time delay since the peak_time when blood flow decays below of the half peak_flow value.
- The Renormalization_time: is the total delay until the reperfusion flow returns to its steady state (Flow0).
- AUC_Reperfusion_Flow = AUC of the first 60 s of the reperfusion phase.
- **Flow_ratio** = Peak_flow/Flow0
- AUC_Reperfusion_Flow = AUC_Reperfusion_Flow/ Flow0_AUC
- Other parameters were not considered in this study

Prior to this study, the E4-diagnose device (Polymath Company. Tunisia) and the different above parameters underwent a heavy validation process in comparison to other reference techniques. In a cohort of 33 patients with lower limb arterial occlusive disease, we compared E4-diagnose parameters to a Vendys 5000 (Endothelix, Inc., USA) an FDA-approved device for digital thermal monitoring. Both devices are sensing skin temperature with different sensors, occlusion sites (brachial cuff in Vendys), target occlusion pressures and totally different calculation algorithms. Both devices shared the same limitations, mainly cold finger index, unsteady room temperature,etc. While Vendys gave vascular reactivity index (VRI) as a unique index, E4-diagnose offered many other parameters and particularly skin blood flow and derived indices from the laser doppler and near infrared spectrometry fields. Comparison of different parameters yielded the best accordance between VRI and EQI, mean VRI 1.33 vs EQI 1.11 (p=0.16), Pearson’s r = 0.836 (p<10^-4^) (**Supplementary figure S2**).

# Supplementary Figures and Tables

## Supplementary Figures


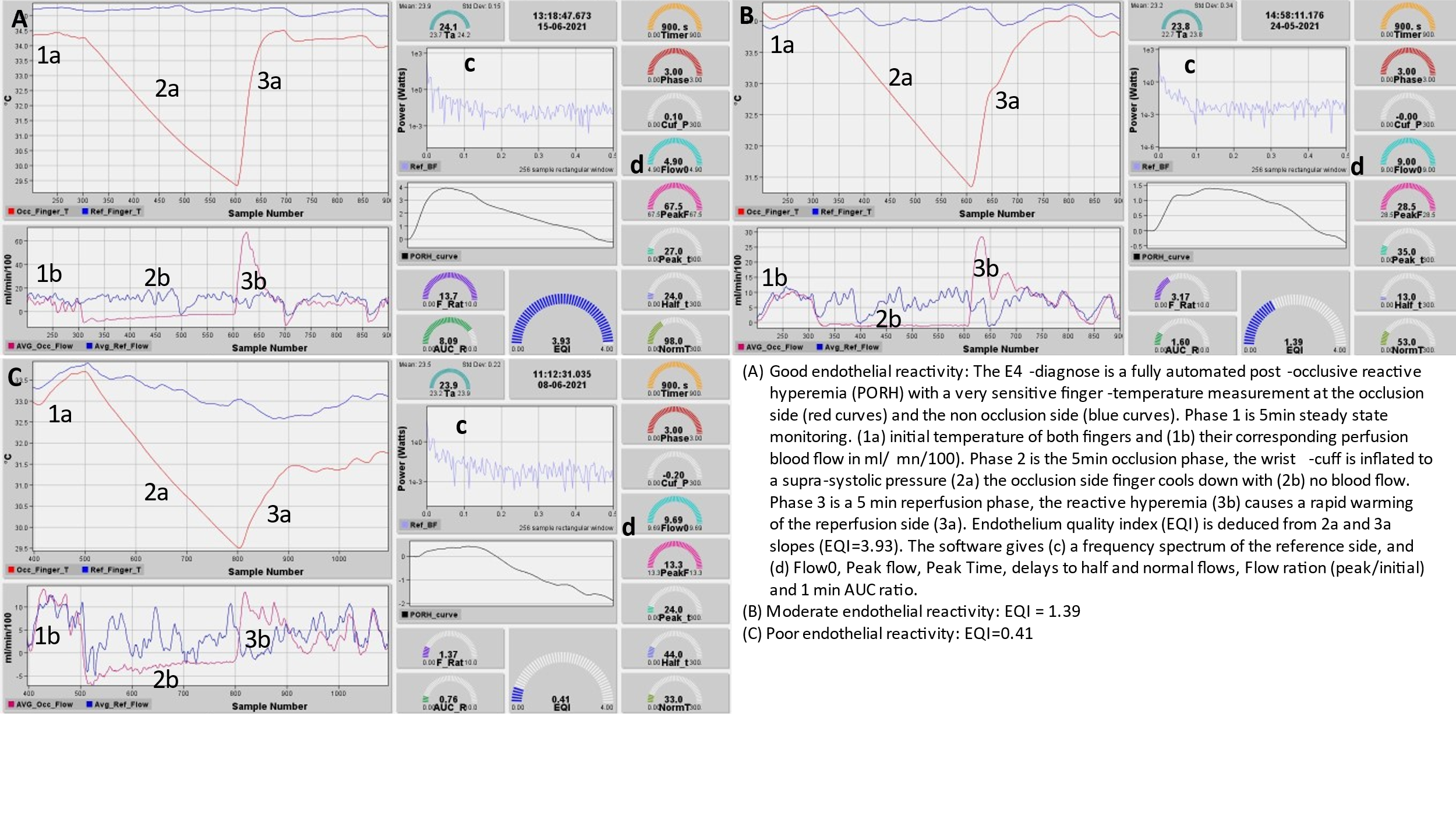


**Supplementary Figure 1.** Different endothelial function parameters assessed by the E4-diagnose device (Polymath Company. Tunisia)


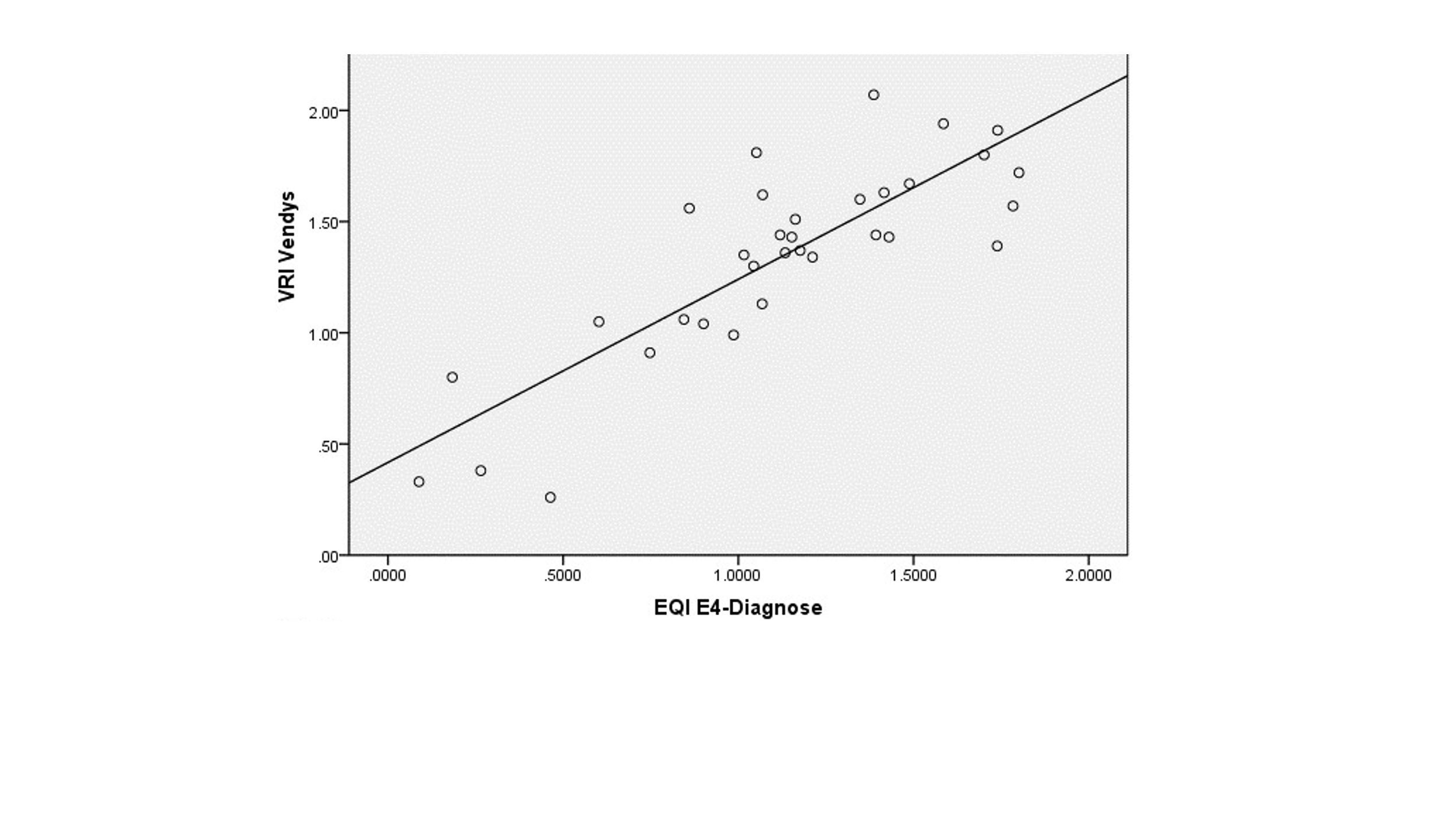


**Supplementary Figure 2:** Correlation between vascular reactivity index (VRI) of Vendys 5000 (Endothelix, Inc., USA) and Endothelium Quality Index (EQI) of E4-diagnose device (Polymath Company. Tunisia).


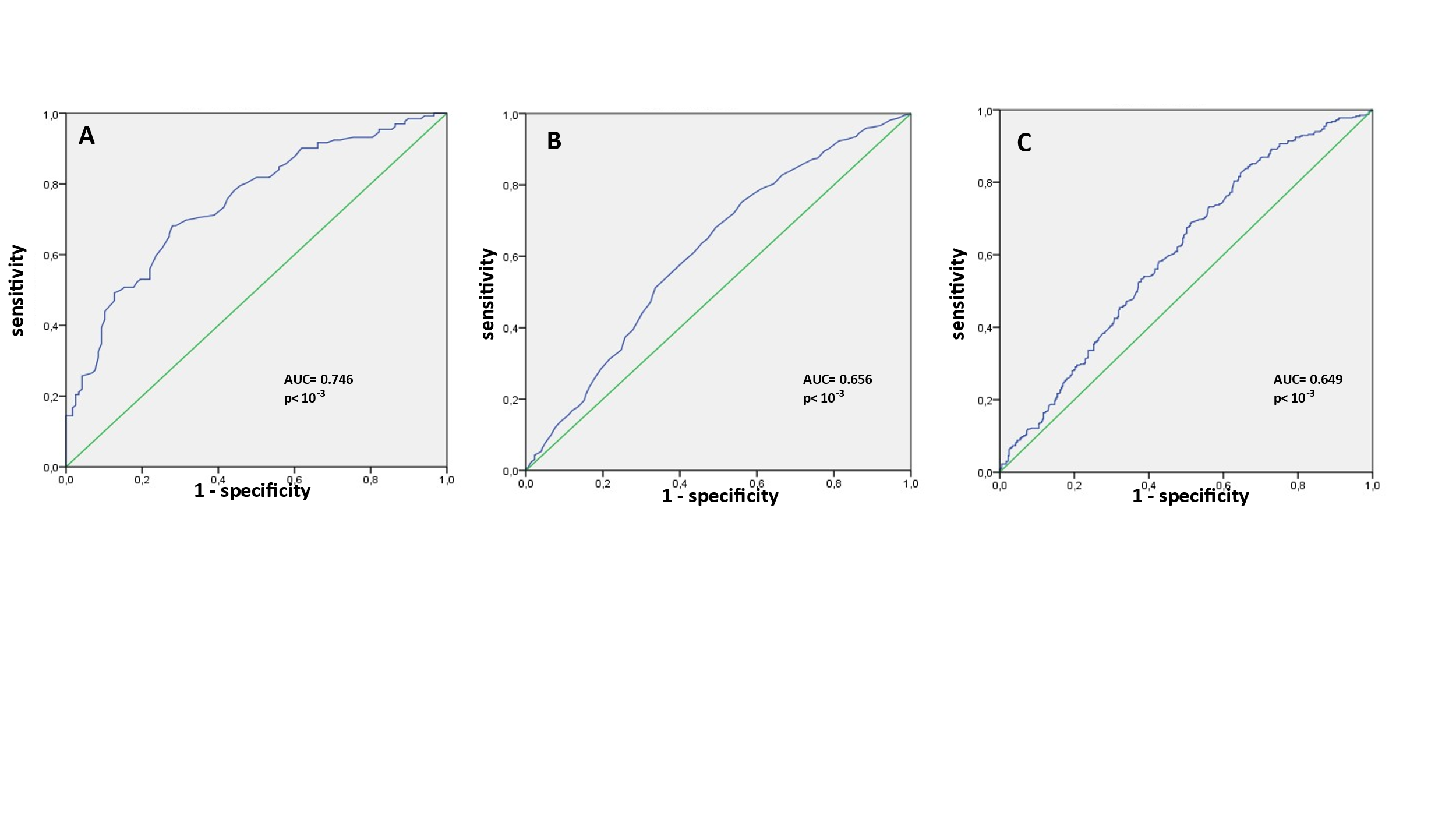


**Supplementary Figure 3.** ROC analysis for A. left ventricle global longitudinal strain, B. Age, C. Body mass index
